# Plasma Brain Natriuretic Peptide Levels in Children with Chronic Kidney Disease and Renal Transplant Recipients: A Single Center Study

**DOI:** 10.3390/children9060916

**Published:** 2022-06-19

**Authors:** Anastasia Garoufi, Aikaterini Koumparelou, Varvara Askiti, Panagis Lykoudis, Andromachi Mitsioni, Styliani Drapanioti, Georgios Servos, Maria Papadaki, Dimitrios Gourgiotis, Antonios Marmarinos

**Affiliations:** 1 Lipid Outpatient Unit, 2nd Department of Pediatrics, Medical School, National and Kapodistrian University of Athens (NKUA), “P. & A. Kyriakou” Children’s Hospital, Thivon & Levadias Str., 11527 Athens, Greece; angaruf@med.uoa.gr (A.G.); steldra@hotmail.com (S.D.); papadaki.mairh@gmail.com (M.P.); 2 2nd Department of Pediatrics, “P. & A. Kyriakou” Children’s Hospital, 11527 Athens, Greece; akoumparelou@gmail.com; 3 Department of Nephrology, “P. & A. Kyriakou” Children’s Hospital, 11527 Athens, Greece; vaskiti@gmail.com (V.A.); amitsioni@icloud.com (A.M.); 4 Honorary Lectures Division of Surgery of International Science, University of College London (UCL), Gower St, London WC1E 6BT, UK; p.lykodudis@ucl.ac.uk; 5 Pediatric Cardiology Unit, “P. & A. Kyriakou” Children’s Hospital, 11527 Athens, Greece; servosge78@gmail.com; 6 Laboratory of Clinical Biochemistry—Molecular Diagnostic, 2nd Department of Pediatrics, Medical School, NKUA, “P. & A. Kyriakou” Children’s Hospital, 24 Mesogeion Avn, 11527 Athens, Greece; dgourg@med.uoa.gr

**Keywords:** hemodialysis, pre-dialysis stage, end-stage renal disease, diastolic dysfunction

## Abstract

Pediatric chronic kidney disease (CKD) patients, as well as kidney transplant patients, are at an increased risk of developing cardiovascular disease. BNP measurement, as a biomarker of cardiovascular risk, has been recommended to this high-risk population. Plasma BNP levels were measured in 56 CKD children in either pre-dialysis stage, hemodialysis (HD) or renal transplant recipients (RTRs) and in 76 sex- and age-matched healthy controls. BNP levels were investigated in HD children, before and after the completion of their HD session. BNP levels in total CKD population, in pre-dialysis stage patients and on HD were significantly higher, compared to the respective controls. HD children had higher BNP levels compared to CKD patients in the pre-dialysis stage. Moreover, post-HD BNP concentration was slightly higher than pre-HD, with the difference being marginally statistically significant. BNP was positively correlated with eGFR, creatinine, cystatin-C and parathormone and negatively with albumin and 25-hydroxyvitamin D. A positive correlation between BNP concentration and the ratio of E/A in pulse-wave Doppler echocardiography was also observed. In conclusion, CKD pediatric patients, mainly those undergoing HD, have high plasma BNP levels which do not decrease after the HD session. This is indicative of a greater risk for future cardiovascular disease.

## 1. Introduction

A total of eleven to thirteen percent of the overall population of the world, suffers from chronic kidney disease (CKD) [1]. In European countries, pediatric CKD ranges from 11–12 per million of the age-related population (pmarp) and eight pmarp for CKD stages 3–5 and 4–5 respectively, with a male to female ratio of about 1.3 to 2 [2]. Pediatric CKD is associated with increased morbidity and mortality; in children with CKD stage 5, the mortality is 30 times higher than in their healthy peers, while a significant mortality rate is also observed in renal transplant recipients (RTRs) [2].

Cardiovascular disease (CVD), being a major complication of CKD, is the most common cause of death in pediatric CKD, with the risk being 1000 times higher in CKD stage 5 children, compared to healthy children of similar age [3,4]. CVD is responsible for 45% of deaths in children and adolescents under 20 years old who are on renal replacement therapy [4]. According to the American Heart Association (AHA), children and adolescents with CKD or renal transplantation, are included in the same high-risk category for the development of CVD, with children suffering from familial hypercholesterolemia or having undergone heart transplantation [5].

Diastolic dysfunction (DD) and left ventricular hypertrophy (LVH) are the most frequent cardiac complications in pediatric CKD [4]. LVH develops even in children with mild to moderate CKD, with one third of them having an increased left ventricular mass index (LVMI) [4,6]. In dialyzed pediatric patients the prevalence of LVH reaches up to 82% and remains high after renal transplantation. In addition, CKD patients, even RTRs, present significant atherosclerotic lesions [4]. In RTRs, although the renal function has been improved, the vascular remodeling may progress, due to the long-time exposure to cardiovascular risk factors, and also to the use of corticosteroids and other immunosuppressive drugs [4,7].

Brain natriuretic peptide (BNP) is mainly secreted by ventricular myocytes after an increase in left ventricular wall stress, as it may happen in cardiac, renal and liver failure [8]. BNP and its amino-terminal cleavage product, N-terminal pro-B-type natriuretic peptide (NT-proBNP), have been extensively studied in adults and are recommended by international guidelines as classical biomarkers for the diagnosis, prognosis and therapeutic monitoring in patients with cardiac diseases, especially those with heart failure (HF) [9]. In patients with CKD, even those without heart failure (HF), as glomerular filtration rate (GFR) decreases there will be an increase in natriuretic peptides (NPs). In these patients, elevated BNP levels may be the result of an increased cardiac release due to blood volume increase, blood pressure elevation and cardiac hypertrophy, but also is partly due to the impairment clearance from the kidneys [7,10]. In adult CKD patients, the high levels of BNP strongly predict CV events and all-cause mortality, whereas patients with lower levels of BNP during treatment show a better prognosis than those with no change or raised BNP [10].

Similarly, in children, the measurement of blood BNP (or NT-proBNP) has progressively gained wide consensus as a useful and reliable biomarker for the diagnosis, management and probably for prognosis of significant structural and functional CVD [8,11]. As there are data that show an association between BNP levels and CV risk in pediatric CKD patients, the use of BNP as a biomarker of CV risk on a routine basis, has been recommended for this high-risk population [7]. A significant relationship of BNP and NT-proBNP levels with various echocardiograph indices of diastolic ventricular dysfunction and left ventricular hypertrophy (LVH) has been reported in pediatric CKD patients [12,13]. Furthermore, that both BNP and NT-proBNP can be used as an inexpensive screening tool for CKD children with an abnormal heart who require further evaluation and subsequent treatment has been supported [12]. Notably, as BNP has mainly pre-renal clearance and its concentration in CKD patients is less affected by GFR compared to NT-proBNP, there is no need to adjust its cut-off value in patients with early CKD stages. However, in children with CKD stage 5, the cut-off values are expected to differ because of reduced renal excretion and concomitant chronic volume overload [10].

The present study is aimed at the assessment of plasma BNP levels in children and adolescents with CKD in pre-dialysis stage, on hemodialysis (HD), and in renal transplant recipients (RTRs), as well as its relationship with CKD stage, and biochemical, echocardiograph and anthropometric parameters.

## 2. Materials and Methods

### 2.1. Study Design

The current study is a single-center prospective study, carried out at the second Department of Pediatrics of the National and Kapodistrian University of Athens and at the Department of Nephrology of the “P. & A. Kyriakou” Children’s Hospital. The study was approved by the hospital’s Ethics Committee and a written informed consent was received from all participants’ parents or from individuals older than 18 years old, before their enrollment in the study.

### 2.2. Study Population

The study population consisted of 56 children and adolescents, aged 2.8 to 20 years old, with CKD stages 2–5 and 76 sex- and age-matched healthy controls. The most frequent causes of CKD were congenital anomalies of kidney and urinary tract (CAKUT) (*n* = 36, 64.3%), followed by hereditary nephropathies (*n* = 7, 12.5%) and glomerulonephropathies (*n* = 7, 12.5%). Other renal diseases were observed in four children (7.1%) whereas in two patients (3.6%) the cause of CKD was uncertain. At the time of evaluation, 24 out of 56 CKD patients were in pre-dialysis stage, 14 were under chronic HD and 18 were RTRs. A total of 12 out of 14 HD patients were under conventional hemodialysis thrice weekly and the remaining two patients four times per week. Every session lasted approximately 4.5 h. Gambro AK 2005 and Nikisso model dbb 05 were used for dialysis. Dialyzers were chosen according to body surface area (BSA) and low-flux membranes were used. The RTRs patients were under prednisolone and mycophenolate mofetil in combination with cyclosporine or tacrolimus. There were thirty–three (60%) of the total CKD population that were under antihypertensive therapy at the time of evaluation. Inclusion criteria consisted of the initiation of HD sessions and performance of renal transplantation, at least 2 and 6 months before the time of evaluation, respectively. Patients in pre-dialysis stage with CKD stages 1 and 2, on peritoneal dialysis (PD), as well as patients with congenital heart diseases, pulmonary disorders or liver dysfunction and current infections, were excluded from the study.

### 2.3. CKD Definition and Staging Classification

The criteria recommended by the Kidney Disease Quality Outcomes Initiative (K/DQOI) were used for the definition of CKD and the estimated GFR (eGFR) according to the KDQOI CKD classification for CKD staging [14]. In all patients, including RTRs, eGFR was calculated by the Schwartz formula [eGFR = k × (height in cm)/serum Cr (mg/dL)] in mL/min/1.73 m^2^ [15].

### 2.4. Patient Evaluation

In all participants (patients and controls) a complete physical examination was carried out and a morning fasting blood sampling was obtained. In CKD patients, a detailed history of renal disease was recorded, including age of onset, disease etiology, medication use, duration of HD and transplantation.

#### 2.4.1. Anthropometric Measurements

Baseline demographic data, including age (years), body weight (kg), height (cm), body mass index (body weight in kg per height in m^2^) and body surface area (m^2^) were collected. The body weight (BW) and height (Ht) were measured to the nearest 0.1 kg with an electronic scale (SECA) and to the nearest 0.1 cm with a wall stadiometer (Hyssna), respectively, with the subjects lightly dressed and barefoot. The standard deviation scores (z-scores) of BW, Ht and body mass index (BMI) were also calculated according to a standardized age- and sex- specific calculator. In HD patients, the “dry weight” was determined clinically. Blood pressure (BP) was measured thrice with a cuff size suitable for their arm and the child in a sitting position, using an electronic automatic oscillometric device (Dynamap). An average value of three measurements of systolic (SBP) and diastolic BP (DBP) equal or above the 95th percentile, according to chart percentiles for age, sex and height, was defined as hypertension [16]. Standard deviation of SBP and DBP Scores, were also calculated with the use of a standardized calculator based on age, sex and height. In HD children, body weight and blood pressure were measured twice, before and after the completion of HD.

#### 2.4.2. Cardiac Evaluation

A cardiac evaluation was performed in all patients at the time of the enrollment into the study. HD patients were evaluated after the end of the HD session. M-mode, 2D-echocardiography and pulse–wave Doppler were carried out by a senior pediatric cardiologist. M-mode and 2D calculations were carried out with the use of suitable 3.5 and 5.5 MHz probes for the children’s age, in a lying position and after rest, by a Siemens Acuson Sequoia Ultrasound Machine. The left ventricular diameter in the end diastole (LVEDD in cm) and in the end systole (LVESD in cm), posterior wall thickness in diastole (PWT in cm), inter-ventricular septum thickness in end diastole (IVS in cm), left atrial size (LA in cm) and ejection fraction (EF%) were measured. An EF ≤ 55% was used to assess systolic dysfunction [17]. Measurements of IVS, PWT and LVEDD were used to calculate left ventricular mass (LVM in g) according to the Devereux formula: LVM (g) = 0.8 × 1.04 × [(IVSd + LVd + PWd)3 − LVD3] + 0.6 g [18]. LVM was indexed to height and expressed in g/m 2.7 (LVMI) in order to minimize the effect of age, sex, and obesity [19]. LVMI values equal or above the 95th percentile for sex and age-specific reference intervals for healthy children were used to diagnose left ventricular hypertrophy (LVH) [20]. Relative wall thickness (RWT) was also calculated by the equation: RWT = (2 × PWT)/LVEDD. RWT ≥ 0.42 was indicative of concentric hypertrophy and RWT < 0.42 of eccentric hypertrophy [21]. Pulse-wave Doppler echocardiography was used to measure mitral valve early diastolic flow velocity (E in m/sec), late atrial filling velocity (A in m/sec) and deceleration time of E wave (DT in m/sec). The ratio between E and A (E/A) was calculated and a value E/A < 1 was regarded as grade 1 diastolic dysfunction (abnormal relaxation) [17]. Additionally, age- and sex-related standard deviation scores (z scores) of echocardiograph parameters were calculated, with the use of the Boston Children’s Hospital z-score system (https://zscore.chboston.org/ accessed on 23 November 2021) and the Canadian Society of Echocardiography calculator (http://csecho.ca/mdmath/ accessed on 23 November 2021).

#### 2.4.3. Laboratory Data

All participants had venous blood samples collected from them in the morning (8–9 a.m.), after an overnight fast. Routine laboratory investigations included serum urea, creatinine, cystatin C, uric acid, albumin, electrolytes, aspartate aminotransferase (AST), alanine aminotransferase (ALT), gamma-glutamyltransferase (γGT), electrolytes and lipid profile, a complete blood count, plasma intact PTH (iPTH) and serum total 25-hydroxyvitamin D (25(OH)D). In HD children the markers of renal function and electrolytes were measured twice, before and after dialysis. Estimated GFR was also calculated pre- and post–HD. Anemia was defined when the levels of hemoglobulin fell below the lowest normal for age and sex limits.

#### 2.4.4. BNP Measurement

BNP was measured in plasma samples, obtained after centrifugation (1600× *g* for 10 min at 4 °C) of 2 mL whole blood collected in K2EDTA (ethylene diamine tetra acetic acid) tubes which were immediately placed and transported on ice. The obtained plasma was transferred to Eppendorf tubes and stored at −70 °C until the time of the analysis. Plasma BNP Fragment (BI-20852W) was measured by ELISA according to manufacturer’s instructions. The kit came by Biomedica Gruppe (A-1210 Wien, Divischgasse 4, Wien, Austria). The intra- and inter-assay variation of the kit were CV 6% and 8% respectively, and the detection limit was 171 pmol/L (0.171 pmol/mL). In HD patients, the BNP levels were measured twice, before and immediately after the end of HD session.

### 2.5. Statistical Analysis

For Data analysis, the IBM SPSS Statistics v25 software (International Business Machines Corporation, Armonk, NY, USA) was used. The main examined parameter, BNP, was tested for normality of distribution by means of visual assessment (histogram of frequencies) as well as by means of Kolmogorov-Smirnov test and was found to be non-normally distributed. Consequently, all data were treated accordingly, and non-parametric tests were implemented. Scale variables are presented as medians and interquartile range (IQR). Categorical variables are presented in actual numbers and selected group percentages. Correlation between scale variables was assessed using the Spearman test. Correlation between categorical variables was assessed using the Fisher’s exact test for four-fold tables, and using the chi squared test for variables with more than two categories. Distribution of scale variables across groups of categorical variables was assessed using the Mann—Whitney U test for variables with two categories, and using the independent samples median test for variables with more than two categories. For paired measurements before and after intervention, the Wilcoxon test was implemented. Where applicable two-tailed tests were implemented. For the further assessment of scale variables as confounding factors, the z-score was also calculated and examined. Finally, the independency of correlation between BNP and other parameters was tested using multiple linear regression analysis. A *p* value of <0.05 was considered statistically significant.

## 3. Results

### 3.1. Population Description

Our study population consisted of 56 CKD patients, 34 males (61%) and 22 females (39%), aged 2.8 to 20 years old (median age: 11.6 y IQR: 8.4, 14.6 years) with CKD stages 2–5, of which 24 (43%) were in pre-dialysis stage, 14 (25%) underwent chronic HD and 18 (32%) were RTRs. There was no significant difference in median age between the three patient subgroups (*p* = 0.362). In total population, the median duration of CKD was 9.5 years (IQR: 5.3, 12.3 years). In the subgroup of HD patients, the median time on HD treatment was 0.9 years (IQR: 0.4, 1.8 years), while in the RTRs subgroup, the median time from renal transplantation was 3.8 years (IQR: 0.7, 9.3 years). Thirty–three patients (59%) were under antihypertensive medications, while pre-hypertension and hypertension was observed in 14 (25%) and 7 (12.5%) patients respectively. Low total 25(OH)D (<20 ng/mL) and increased intact-PTH levels (>55 pg/mL) had 23 (41%), and 39 (69.6%) of patients, respectively. Anemia was established in 29 patients (52%). The descriptive characteristics of the total CKD population and patient’s subgroups are presented in Table 1.

Regarding the cardiac parameters, increased RTW indicative of concentric hypertrophy was observed in six patients (three on HD, one in pre-dialysis stage, two RTRs) and of eccentric hypertrophy in one HD-patient. Diastolic dysfunction (E/A ratio < 1) and systolic dysfunction (EF ≤ 55%) were found in three and two patients, respectively. A total of four children, all on HD, had thick IVS. Increased LVEDD, PW, and DT, had one, two and two patients respectively. Only one HD-patient had LVH (increased LVMI). The values of cardiac parameters are shown in Table 2.

### 3.2. BNP Levels in Patient Subgroups vs. Controls

The median plasma BNP concentration was significantly higher in the intervention group compared to the sex- and age- matched healthy control group (*n* = 76, *p* < 0.001), while BMI z-score did not differ significantly between the two groups (*p* = 0.407). Similarly, median BNP was significantly higher in the subgroup of CKD patients in pre-dialysis stage (*p* = 0.030), as well as in the HD subgroup before and after dialysis compared to the corresponding control individuals (*p* < 0.001 and *p* < 0.001 respectively). However, no significant difference was found between the RTRs subgroup and the corresponding controls (*p* = 0.273). The above findings can be viewed in Table 3.

### 3.3. Correlations between BNP Levels and Patient Subgroups

Among the different patients’ groups, the HD group before dialysis had significantly higher plasma BNP levels compared to the CKD group in the pre-dialysis stage (*p* = 0.001), as well as to the transplantation group (*p* = 0.001). Within the group of HD patients, median post-dialysis BNP levels were borderline higher than pre-dialysis levels (3.75 pmol/mL, 2.78–5.17 pmol/mL vs. 3.24 pmol/mL, 2.00–4.75 pmol/mL, *p* = 0.048, Wilcoxon test).

The median age was similar between the three subgroups of CKD patients (*p* = 0.362), therefore it should not be considered as a possible confounding factor. In contrast, the median BMI was significantly lower in HD patients and higher in transplanted patients, and therefore could be a confounding factor. BNP levels varied by BMI, although this variation does not seem to be the only reason for the significant difference in BNP levels between the three subgroups of CKD patients.

In a multiple linear regression model, assessing BNP as the dependent variable and including as independent variables those that demonstrated unifactorial statistical significance, none of the two parameters, BMI or subgroup of patients, retained statistical significance. This can be attributed to the small number of patients and their relative uneven distribution in subgroups. Thus, the role of BMI as a confounding factor could not be conclusively examined.

### 3.4. Correlations between BNP Levels and Demographic Parameters, Laboratory Markers, Medication Use and Cardiac Parameters

**Figure 1:**
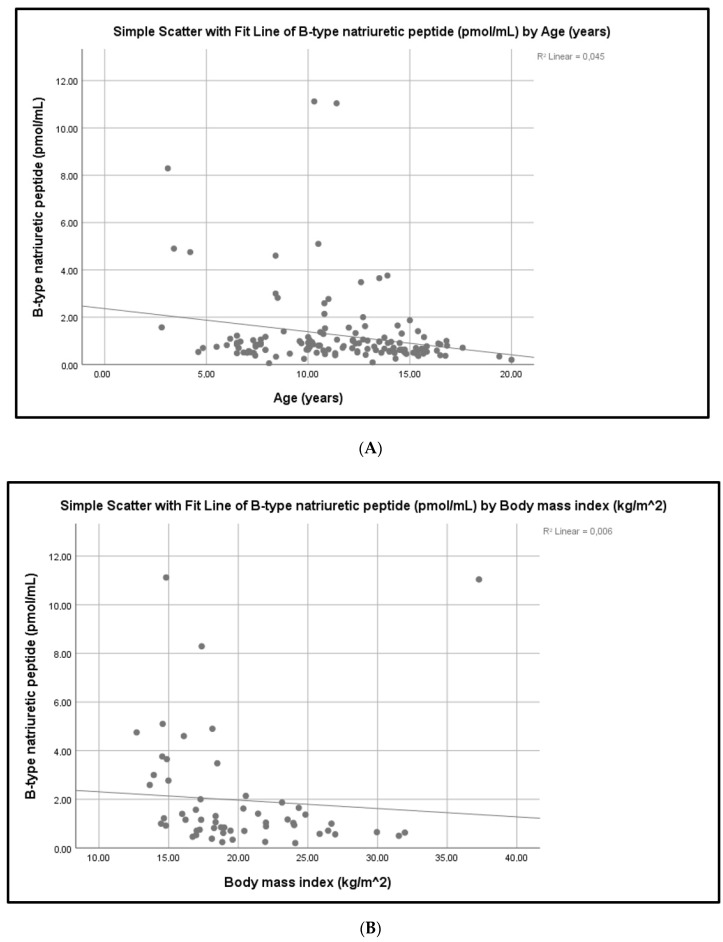
(**A**,**B**). Correlation of plasma BNP levels with age and BMI.

BNP levels were significantly inversely correlated with age (R2 Linear 0.045, *p* = 0.046, Spearman’s correlation) (Figure 1A) and BMI (R2 Linear 0.006, *p* = 0.002, Spearman’s correlation) (Figure 1B).

**Figure 2:**
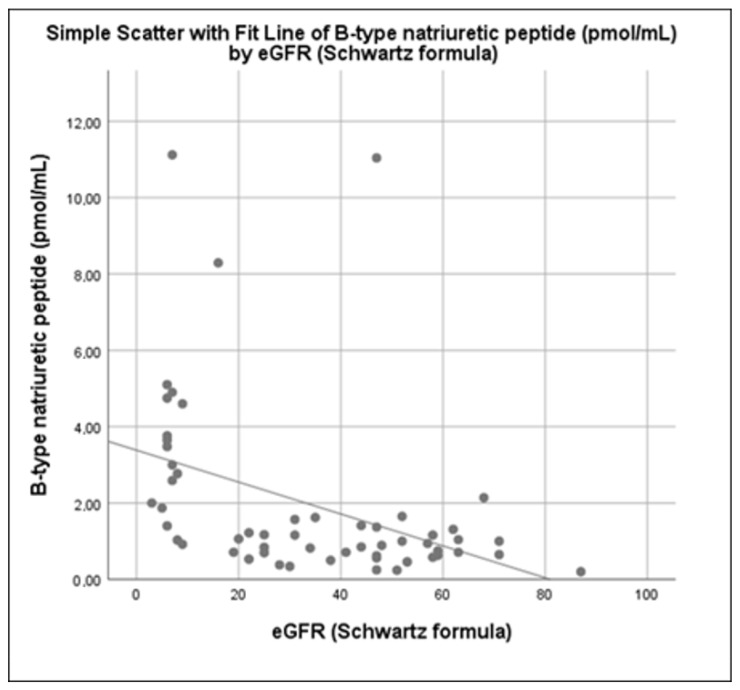
Correlation of plasma BNP levels with eGFR.

In contrary, gender, pubertal stage, duration of CKD and medication use (prednisolone, other immunosuppressive and antihypertensive drugs) were not associated with median plasma BNP levels (*p* > 0.05). A significant inverse correlation was found between plasma BNP levels and eGFR (Figure 2, *p* < 0.001) and a positive one with CKD stage, with the higher levels found in CKD stage 5 (*p* = 0.005).

**Figure 3:**
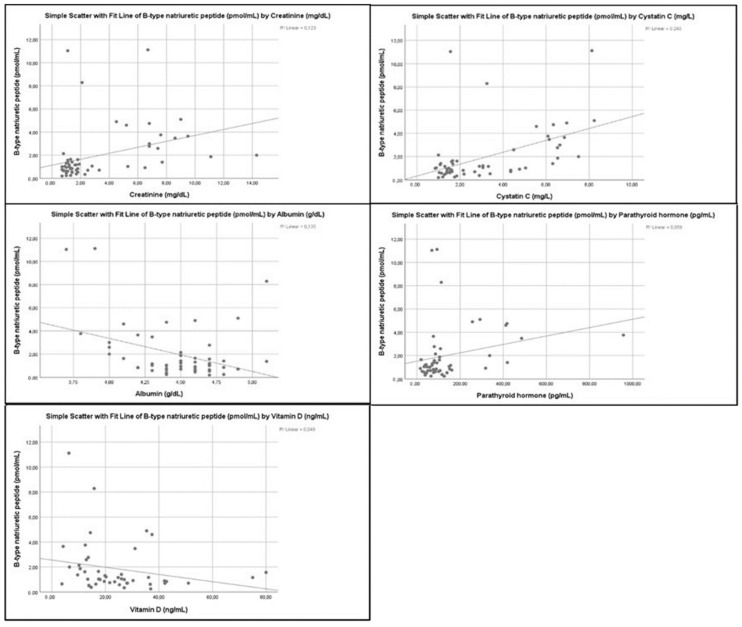
Correlations of plasma BNP levels with biochemical markers.

Moreover, a significant positive correlation exists between plasma BNP and creatinine (*p* < 0.001), cystatin C (*p* < 0.001), as well as with parathyroid hormone levels (*p* < 0.010). BNP was also significantly inversely correlated with 25-hydroxyvitamin D levels (*p* = 0.038), while it had a borderline negative correlation with albumin levels (*p* = 0.05). These correlations are shown in Figure 3.

**Figure 4:**
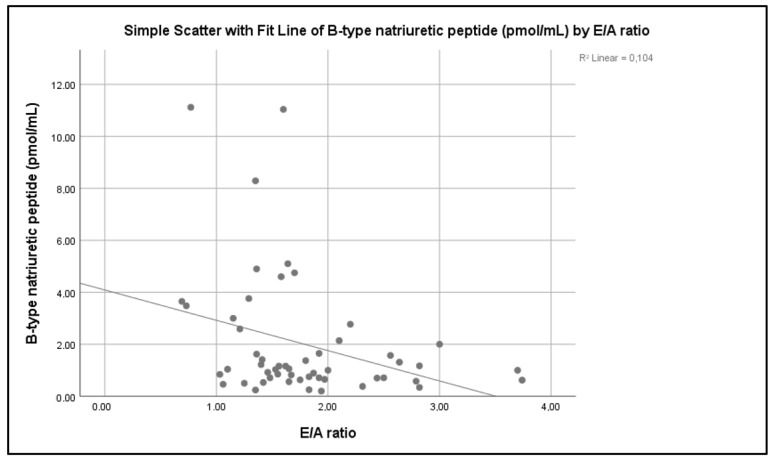
Correlation of plasma BNP levels and E/A ratio.

As far as cardiac and hemodynamic parameters are concerned, only the E/A ratio was significantly inversely correlated with BNP (R2 Linear 0.104, *p* = 0.034 Spearman’s correlation) (Figure 4). There was no statistically significant correlation between BNP levels and EF (*p* = 0.182), LVM (*p* = 0.092), LVMI (*p* = 0.950), SBP (*p* = 0.955), DBP (*p* = 0.230) or HR (*p* = 0.113).

Finally, no significant correlation was found between BNP levels and CRP (*p* = 0.860), presence of dyslipidemia (*p* = 0.376) or anemia (*p* = 0.431). Multiple linear regression analysis was carried out, including BNP as the dependent variable and all parameters which in univariate analyses demonstrated statistically significant correlation with BNP (age, eGFR, BMI z-score, E/A ratio, creatinine, cystatin C, albumin, iPTH and total 25(OH)D). The stronger independent predictor of BNP levels was cystatin C, followed by creatinine, albumin and total 25(OH)D (Table 4). All other variables lost their significance.

Interestingly enough, eGFR per se was not highlighted as an independent prognostic factor for BNP, based on the above model. This could potentially be due to the fact that parameters such as cystatin C and creatinine can be indirect indicators of CKD and therefore various levels of eGFR. Hence, multiple linear regression was run again, this time including as independent variables eGFR, age, BMI z-score, albumin, PTH, 25-hydroxyvitamin D and E/A ratio, as these were considered clinically relevant parameters. In that model, eGFR was indeed demonstrated as an independent prognostic factor. In fact, it was the only factor that maintained correlation (coefficient: −0.029, *p* = 0.03). However, it has to be mentioned that this model was characterized by low accuracy (24%) which means that there is a large proportion of the variability of BNP that aforementioned models cannot predict. Potential reasons for that are the relatively small number of patients and the lack of values at the extremes.

## 4. Discussion

In the current study, the overall CKD and RTRs patients had significantly higher median plasma BNP levels, compared to the healthy sex- and age-matched controls. Comparing the three subgroups, dialyzed, no-dialyzed and RTRs, with their respective controls, only the first two subgroups had BNP levels significantly higher than their controls. Patients undergoing HD had higher BNP concentrations than patients in the pre-dialysis stage. BNP levels and CKD stage display a significant correlation, with CKD stage 5 patients having higher levels. Pre-dialysis BNP levels in HD patients were moderately significantly lower than post-dialysis ones. BNP concentration was significantly positively correlated with serum creatinine, cystatin C, and parathyroid hormone levels and negatively with eGFR, albumin and 25-hydroxyvitamin D levels. Concerning cardiac parameters, only the E/A ratio had a significantly inverse correlation with plasma BNP levels. In multiple linear regression analysis, iPTH and the E/A ratio lost their significance.

Similar to our results, Hedving et al., showed significantly higher BNP levels in pediatric CKD patients undergoing HD and in pre-dialysis stage compared to healthy controls, whereas BNP levels of RTRs did not differ from those of healthy controls [22]. The levels of NT-pro-BNP have also been found to be higher in children on HD and on peritoneal dialysis (PD) than in controls, with HD patients having higher levels than PD ones. In the same study, no significant difference in NT-proBNP concentration was observed between CKD children in pre-dialysis stage and healthy controls [23]. Increased pre-dialysis NT-proBNP concentration in CKD children on HD compared to the controls has also been reported by others [13].

Regarding the pre- and post-dialysis BNP levels, we found a moderate significant increase in median BNP levels (*p*=0.048) immediately after the completion of the low-flux membrane dialysis session. Other studies, both in HD adults and pediatric patients, have shown conflicting results. A decrease, no change or increase in BNP or NT-proBNP levels have been reported [13,23,24,25]. Increased BNP levels 30 min before the HD session and a variable decrease or increase in its levels 30 min post-HD were observed in 33 asymptomatic children with CKD stage 5, with the change not being significant. The authors also reported that pre- and post-dialysis BNP levels were independent predictors of adverse outcome [24]. Similarly, a recent study showed that after a single HD session, there were no significant changes in the plasma NT-proBNP values. According to the authors, this may be due to the fact that dialysis sessions are often shorter than expected, and as a result patients fail to achieve normal blood volume [23]. In contrast, a significant reduction in BNP levels measured immediately after high-flux HD session was found in 30 CKD children. This reduction was interpreted as heart unloading and peptide removal by filtration [25]. Similarly, higher NT-proBNP levels before the initiation of HD compared to those 30 min after the end of HD session, using low-flux membrane dialyzers, have been reported in CKD children. In addition, HD patients with LV dysfunction had significantly higher NT-proBNP levels, both before and after HD, compared to those without LV dysfunction [13]. Finally, studies in adult CKD patients undergoing HD have also shown a decrease, no change or increase in BNP or NT-proBNP levels after the end of the HD session [26,27,28].

A variety of factors have been attributed to the contradictory results of the studies, including the type of NP measured (BNP or NT-proBNP), the type of the dialyzer used in HD (low-flux or high-flux membranes), and the number of hours spent undergoing dialysis each week [29,30]. BNP has a smaller molecular weight and a shorter half-life compared to NT-pro BNP [31]. High-flux membranes display a higher ultrafiltration rate than low-flux ones, resulting in a significant reduction in BNP concentration after high-flux membrane dialysis and a smaller one after low-flux membrane dialysis [29,32]. Regarding NT–proBNP, the authors reported a significant decrease after high-flux membranes HD and an increase after low-flux membranes HD [29,32]. Finally, the time of blood collection, in relation to the HD session, as well as the measurement of peptides in blood serum or plasma in different studies, may also affect BNP and NT-proBNP levels. In general, the potentially confusing effect of HD sessions on blood BNP or NT-proBNP levels, needs to be taken into consideration [30].

Our study population showed an inverse correlation between BNP levels and both age and BMI. In contrast, BNP levels showed no association with either gender or pubertal stage. In general, age, gender, pubertal status and BMI have been reported to modify plasma BNP levels [7]. Infants and children have lower BNP levels than adults [33]. BNP concentrations remain steady after the first month of life, without any significant change up to the 10–12 years of age mark [8,33]. During adolescence, BNP levels increase significantly [7,33]. BNP levels in children, showed no gender-related differences to BNP levels, up to the beginning of adolescence. [7,8]. As adolescence progresses, BNP levels gradually increase, with girls having higher levels than boys. This may be due to the direct positive effect of female steroid hormones and the negative effect of male sex hormones on BNP production by cardiomyocytes [8]. This sexual difference persists in adulthood. [7]. In contrast, another study in healthy children showed no gender or age-related differences in NT-proBNP levels [34]. Finally, a negative association of BNP levels with BMI has been observed in most studies, both in healthy subjects and patients with CV diseases [7,35]. In contrast, no correlation between BMI and NT-proBNP levels in HD patients has been reported [36].

The different results of the studies referring to the normal levels of BNPs may be due to methodological differences since BNPs values are method dependent [11]. Furthermore, the existing different pathophysiologic stimuli and cardiovascular hemodynamics, both in healthy individuals and in patients with heart failure (HF), can be responsible for the wide fluctuation of plasma BNP concentrations [8].

A significant inverse correlation was found, between BNP levels and eGFR and a positive one between BNP levels and CKD stage, serum creatinine and cystatin C levels. An increase in BNP or NT-proBNP levels with decreasing eGFR and increasing CKD stage has been reported in other studies [10,12,36,37]. In a study by Rinat et al., eGFR was an independent factor influencing BNP and NT-proBNP values [12]. Similarly, decreased eGFR was independently associated with elevated NT-proBNP levels in RTR children and young adults [38]. A significant positive correlation between BNP (or NT-proBNP) levels and renal function parameters such as creatinine and cystatin C, has also been reported [36,39].

We did not observe any correlation between BNP levels and anemia. Similarly, in a study in HD children, NT-proBNP levels were not correlated with anemia [36]. In contrast, a significant negative correlation of BNP or NT-prοBNP and anemia has been found in several studies [12,22]. Moreover, Hedvig et al., showed that BNP concentration >100 pg/mL had a high predictive value for the incidence of anemia [22].

In the current study, multiple linear regression analysis showed that low albumin and 25(OH)D levels were independent predictors of high BNP levels. In contrast, a positive correlation between BNP and iPTH levels was observed only in unifactorial analysis. A negative or no correlation of BNP (or NT-proBNP) with 25(OH)D levels, have been reported by others [40,41]. An inverse correlation between BNP or NT-proBNP and albumin levels has been reported in patients with cardiac heart failure and poor long prognosis [42]. Finally, higher BNP levels in CKD patients with high iPTH than those with low iPTH, as well as no relation between the above parameters, have been reported [22,36,37,41].

Data concerning the relationship between BNP and indices of functional and morphological cardiac abnormalities are also conflicting. A significant association between BNP levels and heart geometry has been reported in pediatric CKD patients, with elevated BNP levels being a predictor of abnormal heart geometry [12,22]. Notably, studies support that both BNP and NT-proBNP could serve as surrogate markers of myocardium stress in pediatric CKD stages 3–4 patients, despite the fact that both peptide levels are affected by GFR [12,22].

In the present study, there was no correlation observed between BNP levels and LVMI, in all patient groups. Similarly, no association between NT-proBNP and LVMI or volume overload has been observed by others in CKD children in pre-dialysis stage [43]. In contrast, other studies have found a positive correlation between BNP or NT-proBNP levels and LVMI in CKD children as well as in RTRs [22,23,38,44]. Our study showed that the E/A ratio, a marker of diastolic function, had a significant inverse correlation with BNP. The significance was not maintained in multiple linear regression analysis, for the assessment confounding factors. Many studies report a correlation between BNP and NT-proBNP levels and indices of diastolic dysfunction in CKD children [12,13,23,44]. Moreover, routine measurement of BNP in CKD children on PD, in order to evaluate the risk of functional and morphological cardiac abnormalities, has been recommended [44].

In a recent study on pediatric patients undergoing HD, echocardiograph parameters were assessed before and after HD session. Pre- and post-HD BNP levels were positively correlated with different echocardiographic variables, mirror LV diastolic function, before and after HD session, respectively. A significant reduction in LV and LA diameters, as well as of trans mitral E velocity and the E/A ratio after HD session was reported [25]. A positive correlation between pre-HD BNP levels and DT has also been reported [24].

Concerning other cardiac and hemodynamic parameters, our study showed no significant associations between BNP levels and EF, SBP or DBP. Again, bibliographic data remain controversial in the matter. A negative correlation between EF, a marker of systolic dysfunction, and BNP or NT-proBNP has been reported by others [13,24]. In contrast, in a recent study on HD children, EF was not affected by HD and its values were not correlated to pre- and post-HD BNP levels [25]. Finally, although there are data supporting a positive correlation between BNP (or NT-proBNP) levels and systolic and/or diastolic blood pressure in CKD children, other studies showed, in accordance with our results, that NT-proBNP levels did not correlate with SBP or DBP [12,13,23,37,38].

In a previous study from our team, that used the same sample pool as the present study, concerning urotensin II (UII) levels, another prediction marker of CVD in CKD patients, showed that CKD children in pre-dialysis stage and RTRs had significantly higher levels than healthy subjects. Moreover, whereas UII levels in HD children did not differ significantly from healthy controls before HD, they did increase significantly at the end of the HD session [45].

In conclusion, CKD children and adolescents undergoing HD and those in the pre-dialysis stage have increased plasma BNP levels, whereas RTRs have BNP levels similar to age- and sex- matched healthy controls. HD patients (CKD stage 5) have the highest BNP levels, which do not decrease after the end of the HD session. The renal function markers, such as creatinine and cystatin C, are independent predictors of high BNP levels. BNP was also inversely correlated with the E/A ratio, a marker of diastolic dysfunction. The above findings indicate that pediatric CKD patients are at an increased risk for cardiovascular diseases.

## 5. Limitations of the Study

The main limitations of the present study include the relatively small number of CKD children, especially those on HD, and the lack of repeated measurements of the BNP levels. Additional limitations include the assessment of wet weight in HD children that was based only on clinical findings and the cardiac evaluation of the HD children that took place only once, after the end of the HD session.

## Figures and Tables

**Table 1:** Demographic, clinical and laboratory characteristics of CKD patients (*n* = 56).

Parameter	Total (*n* = 56)	Pre-Dialysis Stage Group (*n* = 24)	HD Group (*n* = 14)	RTRs Group (*n* = 18)	*p* Value
Age (years)	11.55 (8.4–14.5)	11.45 (7.9–15.35)	10.65 (8.4–12.7)	12.2 (10.6–14.6)	0.362
Gender M/F (%)	60.7/39.3	70.8/29.2	57.1/42.9	50/50	0.373
Pre-pubertal/Pubertal stage (%)	50/50	50/50	64.3/35.7	38.9/61.1	0.109
CKD stages 2 and 3 (%)	12.5/42.9	0/58.3	0/0	38.9/55.6	
CKD stages 4 and 5 (%)	16.1/28.6	33.3/8.3	0/100	5.6/0	
BMI (kg/m^2^)	18.58 (16.6–20.93)	18.61 (16.74–20.8)	15.97 (14.82–19.2)	19.1 (17.98–21.98)	0.004
BMI z-score	−0.17 (−0.68–0.44)	−0.16 (−0.64–−0.41)	−0.84 (−1.14–0.01)	−0.03 (−0.32–0.71)	0.004
SBP (mmHg)	109 (99–119)	107 (96–120)	111 (87–119)	107 (100–118)	0.084
DBP (mmHg)	67 (56–74)	65 (52–73)	68 (55–77)	68 (61–73)	0.004
Pre-hypertension /Hypertension (%)	25/12.5	16.7/4.2	35.7/28.6	27.8/11.1	0.615
eGFR (ml/min/1.73 m^2^)	33 (9–52)	31 (22–47)	6 (6–7)	59 (47–63)	<0.001
Creatinine (mg/dl)	1.65 (1.1–5.25)	1.7 (1.3–2.2)	7.5 (6.8–9)	1 (0.8–1.2)	<0.001
Cystatin C (mg/L)	2.01 (1.42–4.91)	1.86 (1.55–3.16)	6.54 (6.15–6.96)	1.42 (1.04–1.67)	<0.001
Albumin (g/dl)	4.5 (4.3–4.7)	4.6 (4.5–4.7)	4.3 (4–4.6)	4.45 (4.4–4.7)	0.048
Hemoglobulin (g/dl)	11.85 (10.7–12.9)	12.35 (10.85–13.6)	10.9 (10.5–11.9)	11.8 (11–12.7)	0.131
Hematocrit (%)	35.75 (32.85–38.7)	37.2 (34.05–39.95)	33.75 (31.3–35.7)	36 (32.4–39.6)	0.043
Anemia (%)	51.8	37.5	71.4	55.6	0.121
iPTH (pg/mL)	85.35 (60.69–144)	82.61 (50–116.3)	257 (92.92–416.9)	76.15 (57.65–92.24)	0.066
25(OH)D (ng/mL)	20.24 (13.6–31.06)	28 (19.57–37.4)	12.87 (6.57–14.37)	19.82 (13.89–25.99)	0.033

Scale variables are presented as medians (Q1, Q3). CKD: chronic kidney disease, HD: hemodialysis, RTRs: renal transplant recipients, BMI: body mass index, SBP: systolic blood pressure, DBP: diastolic blood pressure, eGFR: estimated glomerular flow rate, iPTH: impact parathyroid hormone, 25(OH)D: 25-hydroxyvitamin D.

**Table 2:** Cardiac parameters’ values in CKD patients.

Parameter	Total (*n* = 53)	Pre-Dialysis Stage Group (*n* = 24)	HD Group * (*n* = 12)	RTRs Group (*n* = 17)
LA (cm)	2.57 (2.25–2.89)	2.6 (2.15–2.82)	2.29 (2.09–2.57)	2.76 (2.43–3.1)
LVEDD (cm)	3.81 (3.32–4.12)	3.85 (3.4–4.33)	3.18 (2.93–3.62)	3.94 (3.58–4.12)
LVESD (cm)	2.23 (1.83–2.42)	2.26 (1.9–2.61)	1.94 (1.73–2.11)	2.33 (2.11–2.42)
IVSd (cm)	0.66 (0.55–0.74)	0.66 (0.52–0.75)	0.64 (0.57–0.76)	0.62 (0.55–0.73)
PWT (cm)	0.6 (0.55–0.68)	0.58 (0.5–0.66)	0.6 (0.55–0.71)	0.66 (0.57–0.75)
EF (%)	70.4 (65–72.8)	71 (66.25–75.4)	65.15 (62.35–71.45)	70.5 (66.4–72.2)
E (m/sec)	0.85 (0.78–1.03)	0.85 (0.79–1.1)	0.84 (0.52–0.95)	0.87 (0.83–1.03)
A (m/sec)	0.51 (0.43–0.65)	0.51 (0.38–0.6)	0.51 (0.49–0.67)	0.5 (0.4–0.68)
E/A ratio	1.65 (1.36–2)	1.65 (1.46–2.44)	1.33 (0.96–1.67)	1.78 (1.41–2)
DT (m/sec)	158.5 (140–178)	164 (146–182)	162 (140–180)	152 (130–174)
RWT	0.32 (0.29–0.37)	0.31 (0.28–0.33)	0.36 (0.34–0.43)	0.33 (0.29–0.37)
HR (heats/sec)	87 (75–96)	93 (71–105)	91 (80–96)	85 (81–91)

LA: left atrial size; LVEDD: left ventricular diameter in the end diastole; LVESD: left ventricular diameter in the end systole; IVSd: inter-ventricular septum thickness in end diastole; PWT: posterior wall thickness in diastole; EF: ejection fraction; E: mitral valve early diastolic flow velocity; A: late atrial filling velocity; DT: deceleration time of E wave; RWT: relative wall thickness; HR: heart rate. * Post-dialysis measurements.

**Table 3:** Comparison of BNP levels (Median, IQR) between groups and subgroups of the study *.

Groups/Subgroups	BNP (pmol/mL) Median, IQR	*p* Value
Overall CKD Patients	1.05 (0.71, 2.07)	*p* < 0.001
Control group	0.67 (0.51, 091)	
CKD in Pre-dialysis stage	0.91 (0.59, 1.19)	*p* = 0.030
Corresponding Controls	0.65 (0.51, 0.90)	
HD: before dialysis	3.24 (2.00, 4.75)	*p* < 0.001
Corresponding Controls	0.61 (0.44, 0.80)	
HD: after dialysis	3.75 (2.78, 5.17)	*p* < 0.001
Corresponding Controls	0.61 (0.44, 0.80)	
RTRs	0.83 (0.63, 1.31)	*p* = 0.273
Corresponding Controls	0.77 (0.51, 0.97)	

* MWU test; IQR: 25th–75th percentile; BNP: Brain natriuretic peptide; CKD: chronic kidney disease; HD: hemodialysis; RTRs: renal transplant recipients.

**Table 4:** Multiple linear regression analysis * for identifying independent predictors of BNP levels in the total CKD population.

Parameter	Coefficient	*p* Value
Cystatin C	1.075 (0.651–1.500)	<0.001
Creatinine	−0.555 (−0.885–−0.224)	0.001
Albumin	−1.988 (−3.286–−0.691)	0.003
Total 25(OH)D	−0.037(−0.0070–−0.004)	0.028

* Model: Standard model, 95% CI, stepwise forward, information criterion (AICC), based on medians.
